# Involvement of a *Toxoplasma gondii* Chromatin Remodeling Complex Ortholog in Developmental Regulation

**DOI:** 10.1371/journal.pone.0019570

**Published:** 2011-05-31

**Authors:** Peggy J. Rooney, Lori M. Neal, Laura J. Knoll

**Affiliations:** Department of Medical Microbiology and Immunology, University of Wisconsin School of Medicine and Public Health, Madison, Wisconsin, United States of America; Istituto Dermopatico dell'Immacolata, Italy

## Abstract

The asexual cycle of the parasite *Toxoplasma gondii* has two developmental stages: a rapidly replicating form called a tachyzoite and a slow growing cyst form called a bradyzoite. While the importance of ATP-independent histone modifications for gene regulation in *T. gondii* have been demonstrated, ATP-dependent chromatin remodeling pathways have not been examined. In this study we characterized C9, an insertional mutant showing reduced expression of bradyzoite differentiation marker BAG1, in cultured human fibroblasts. This mutant contains an insertion in the gene encoding TgRSC8, which is homologous to the *Saccharomyces cerevisiae* proteins Rsc8p (remodel the structure of chromatin complex subunit 8) and Swi3p (switch/sucrose non-fermentable [SWI/SNF]) of ATP-dependent chromatin-remodeling complexes. In the C9 mutant, *TgRSC8* is the downstream open reading frame on a dicistronic transcript. Though protein was expressed from the downstream gene of the dicistron, TgRSC8 levels were decreased in C9 from those of wild-type parasites, as determined by western immunoblot and flow cytometry. As TgRSC8 localized to the parasite nucleus, we postulated a role in gene regulation. Transcript levels of several markers were assessed by quantitative PCR to test this hypothesis. The C9 mutant displayed reduced steady state transcript levels of bradyzoite-induced genes *BAG1*, *LDH2*, *SUSA1*, and *ENO1*, all of which were significantly increased with addition of TgRSC8 to the mutant. Transcript levels of some bradyzoite markers were unaltered in C9, or unable to be increased by complementation with *TgRSC8*, indicating multiple pathways control bradyzoite-upregulated genes. Together, these data suggest a role for TgRSC8 in control of bradyzoite-upregulated gene expression. Thus chromatin remodeling, by both ATP-independent and dependent mechanisms, is an important mode of gene regulation during stage differentiation in parasites.

## Introduction

*Toxoplasma gondii* is an obligate intracellular parasite capable of infecting a wide range of species, including all warm-blooded mammals tested. This parasite is transmitted primarily by two stages within its lifecycle. The sexual cycle occurs in felines, the definitive host, and results in the shedding of copious environmentally stable oocysts in the feces. The asexual life cycle occurs within intermediate hosts, where the infectious form of the parasite differentiates to rapidly replicating tachyzoites, capable of disseminating through the body. Signals from the host likely trigger conversion to the slow growing bradyzoite form, which is harbored in brain and muscle tissue within cysts. These largely quiescent cysts can remain for the life of the host, and provide the likely route of transmission by which most humans contract the infection, via consumption of undercooked meat [1], [2]. Immune suppression of the host can lead to differentiation to tachyzoites, whose cytolytic properties result in disease in the forms of encephalitis, myocarditis or chorioretinitis.

Although drugs can combat the replicating tachyzoites, no therapy exists to eradicate established bradyzoites. As such, mechanisms of differentiation between tachyzoites and bradyzoites have been the subject of intense research efforts in the hope of isolating key factors as drug targets. These studies are facilitated by the ability to differentiate tachyzoites to bradyzoites in vitro [3]. Several bradyzoite upregulated genes have been identified, shifting the focus towards unraveling modes of stage-specific gene regulation within *T. gondii*. In silico surveys have identified cis-acting elements in the upstream regions of tachyzoite and bradyzoite genes [4], [5]. Likewise, recent genome analyses have highlighted the importance of Apetala2 (AP2) transcription factors in Apicomplexan gene regulation [6], [7]. Understanding the contributions of transcription factors to developmental regulation in *T. gondii* will be an important future area of study.

In *T. gondii*, eukaryotic promoters mechanisms combine with epigenetics to regulate developmental gene expression [4]. As in other systems, *T. gondii* uses modification of histones to regulate gene expression, where acetylation is associated with activated transcription [8]. In tachyzoites, histone acetyl-transferase (HAT) TgGCN5-A acetylates lysine residues in amino-terminal histone tails at the tachyzoite *SAG1* promoter to allow transcription, while histone deacetylase corepressor (HDAC) TgHDAC3 inhibits transcription at bradyzoite-induced *BAG1* and *LDH2* promoters [9]. Another GCN5 homolog and other HATs belonging to the MYST family were also characterized in *T. gondii*, while other HATs and HDACs have been identified within the genome but remain unstudied [7], [10]-[12]. Methylation of histone lysine and arginine residues is associated with activation or silencing of genes, and histone arginine methyltransferases TgPRMT1 and TgCARM1 have been investigated in *T. gondii* [9], [12], [13].

While one class of chromatin remodelers involves the post-translational modifications of histones, another class involves the remodeling of nucleosomes. Chromatin remodeling complexes of the Swi2/Snf2 group, such as the highly similar multiprotein SWI/SNF and RSC complexes, control gene expression by the repositioning of nucleosomes in a manner fueled by ATP hydrolysis. The yeast SWI/SNF complex is recruited to RNA polymerase (Pol) II promoters, while the RSC complex contains several subunits encoded by essential genes, and is recruited to Pol III and specific classes of Pol II promoters [14], [15]. The RSC complex can act in response to stress and target promoters of stress-responsive genes [16]. Members of these complexes have been identified in *T. gondii*, including Snf2-related CBP activator protein TgSRCAP [17]. In this study, we describe characterization of an insertional mutant in *TgRSC8*, a locus showing homology to yeast paralogs Swi3p and Rsc8p of SWI/SNF and RSC complexes, respectively. The insertion led to a reduction but not an elimination of TgRSC8 protein. In the insertional mutant, transcription of housekeeping genes was unaffected, however, TgRSC8 reduction decreased the steady state transcript levels of some but not all bradyzoite-induced genes analyzed. Collectively, these data indicate a role for ATP-dependent chromatin remodeling complexes in the regulation of differentiation-associated genes in *T. gondii*.

## Materials and Methods

### Ethics statement

Animals were housed under conventional, specific-pathogen-free conditions and were treated in compliance with guidelines set by the Institutional Animal Care and Use Committee of the University of Wisconsin School of Medicine and Public Health (IACUC), according to IACUC approved protocol. The University of Wisconsin is accredited by the International Association for Assessment and Accreditation of Laboratory Animal Care.

### T. gondii *growth*

*T. gondii* strains used were derivatives of either Pru, or PruΔ*HPT* (Pru with a deletion in the hypoxanthine-xanthine guanine phosphoribosyltransferase gene (HPT)). Strains were grown as tachyzoites in human foreskin fibroblasts (HFF) maintained in Dulbecco's modified eagle medium containing 4.5 g/L D-glucose, supplemented with 10% fetal bovine serum (FBS), 2 mM glutamine, 100 units/ml penicillin and 100 µg/ml streptomycin at 37°C in 5% CO_2_. Strain C9 is Pru transformed with pT230-Tub5/CAT [18]. Vector control (VC) strains used were transformed with the same vector. VC1 has a plasmid insertion upstream of predicted patatin-like phospholipase *TgPL2* (TgME49_105140 in the draft 6 annotation of the *T. gondii* genome database [ToxoDB], http://toxodb.org) and VC2 was disrupted in TgME49_115700 [19]. In vitro bradyzoites were generated by infecting tachyzoites at 1×10^4^/well in 24 well coverslip plates or 2×10^6^/T-25 to HFF monolayers 3 weeks post-seeding and incubating at 37°C, 5%CO_2._ After 3.5 h, the medium was replaced with RPMI1640 supplemented with 1% FBS and 42 mM *N*-2-hydroxyethylpiperazine-*N'*-2-ethanesulfonic acid (HEPES), 100 units/ml penicillin and 100 µg/ml streptomycin, at pH 8.1, and incubating at 37°C, ambient CO_2_ for 3 days.

### RNA manipulation

RNA was collected from 3 T-25s of intracellular *T. gondii* per sample, using tachyzoites at 50 hours or bradyzoites at 3 days post-infection. Parasites were released by passage through a 27 g needle and collected by centrifugation at 425 x g for 10 min. RNA was isolated using 1 ml Ultraspec (Biotecx Laboratories, Houston, TX, USA) according to manufacturer's directions. RNA was separated on formaldehyde/agarose gels for northern hybridization, used with the FirstChoice RLM-RACE kit (Ambion, Austin, TX, USA) or 10 µg was treated with amplification-grade DNase I and 2 µg was used for cDNA generation by Superscript III First-Strand Synthesis System (Invitrogen, Carlsbad, CA, USA) using random hexamer priming. Probes for northern hybridization include 590 bp of *TgRSC8* amplified from *T. gondii* genomic DNA (gDNA) using RSC8probeF and RSC8probeR (diagramed in Fig. S1), 614 bp of *cat* amplified from pT230-Tub5/CAT using CATprobeF and CATprobeR, and a KpnI-ScaI fragment of *TUB1* digested from pTAT2 [20].

### Plasmid construction

TgRSC8 complementation construct pTPR17 was generated by amplification from genomic DNA (gDNA) of upstream sequences to an EcoNI site within exon 1 using primers P1 with added 5′ EcoNI and SpeI sites and P2 (3 kb; see Table S1, Fig. S1). The coding region from EcoNI site to stop codon, adding a PacI site within the stop, was amplified from cDNA using P3 and P4 (2.3 kb), and the 3′ end was amplified from gDNA using P5 and P6, adding 5′ PacI and 3′ PacI and PmeI sites (1.1 kb). Fragments were cloned in pCR2.1-TOPO (Invitrogen, Carlsbad, CA, USA), and the EcoNI A fragment was added to B. Fragment AB was cloned into the SpeI site of pBC SK+DHFR3, generated by addition of a HindIII-SpeI *DHFR-TS* fragment from pDHFR-TscM2M3 to the NotI site of pBCsk+ (Stratagene, La Jolla, CA, USA), followed by addition of the PacI C fragment [21]. A carboxy-terminal hemagglutinin (HA) tag was added by replacement of the NgoMIV-PacI fragment of pTPR17 with the fragment amplified by P7 and P8 to create pTPR17HA. An amino-terminal HA tag was added using primers pairs P9 and P10, and P11 and P4. The products joined by splicing-by-overlap-extension (SOE) PCR created a 2.9 kb NotI-PacI fragment that replaced the correlary in pTPR17, to create pTPR17NHA. These vectors were linearized with XbaI for electroporation.

Plasmid pTUB1-LUC-5′3′HPT (a gift from G. Arrizabalaga, University of Idaho), containing firefly luciferase (*LUC*) under the control of *T. gondii TUB1* promoter, and 5′ and 3′ untranslated regions (UTRs), was introduced to Pru in replacement of the *HPT* locus by 6-thioxanthine selection [22]. For pBCD3-*cat-TgRSC8-LUC*, the *cat-TgRSC8* fused region was amplified from C9 using primers P16 and P17. A promoterless *LUC* with *T. gondii TUB1* 3′UTR was amplified from pTUB1-LUC-5′3′HPT with P18 and P19. For *cat-LUC*, the cat gene was amplified from pT230-TUB5/CAT using P16 and P20, and *LUC* was amplified using P21 and P19. Amplified fragments were assembled by SOE-PCR, and XbaI fragments containing the fusions were ligated into the SpeI site of pBC SK+DHFR3, and linearized by ApaI for electroporation. The *TgRSC8* coding region was amplified from pTPR17 with P22 and P23, and cloned into NcoI-NotI sites of pET28a+ (Novagen, Merck KGaA, Darmstadt, Germany) to create an *E. coli* expression vector.

Plasmids were introduced to *T. gondii* by electroporations as previously described [23]. Transforming DNA and identification of unique clones with differing insertion sites was determined by Southern hybridization.

### Immunofluorescence assays

Parasites in HFFs on coverslips were fixed in phosphate-buffered saline (PBS)/3% formaldehyde for 20 minutes, neutralized with PBS/0.1 M glycine for 5 minutes, and blocked overnight in PBS/3%BSA/0.2% Triton X-100 at 4°C. *Dolichos biflorus* agglutinin-FITC (Sigma, St. Louis, MO, USA) and antibody reactions and washes were performed in PBS/0.2% Triton X-100 at room temperature. Primary rabbit antisera were provided by I. Coppens, Johns Hopkins Malaria Research Institute, Baltimore, MD (H2Bv), J. Boothroyd, Stanford University School of Medicine, Palo Alto, CA (SAG1) and L.M. Weiss, Albert Einstein College of Medicine, Bronx, NY (BAG1). Murine monoclonal anti-TgRSC8 antibodies were produced for these studies. The TgRSC8 expression vector was transformed into *E. coli* Rosetta (DE3) pLysS (Novagen), and protein was purified from IPTG-induced cultures under denaturing conditions using TALON Nickel affinity resin (Clontech, Mountain View, CA, USA), dialyzed to PBS, and quantified by MicroBCA (Pierce, Rockford, IL, USA). Murine monoclonal antibodies 3AD6 and 1DE10 were developed against this antigen, as previously described [24]. Secondary antibodies included AlexaFluor 488, 633, or 320 anti-mouse or rabbit (Invitrogen, Carlsbad, CA, USA), and coverslips were mounted with Vectashield with or without 4′,6-diamidino-2-phenylindole (DAPI; Vector Laboratories, Burlingame, CA, USA). For localization, images were captured using OpenLab v5.0.1 (PerkinElmer, Waltham, MA, USA), from a 100X objective using Zeiss Axioplan 2 microscope with FluoArc lamp, triple pass (DAPI/FITC/Texas Red) emission cube, differential interference contrast optics, and a Hamamatsu ORCA-AG CCD camera. Resulting images were deconvolved, false-colored and merged using Volocity v4 (PerkinElmer, Waltham, MA, USA). For assessing BAG1 expression, in vitro bradyzoites were incubated with murine anti-*T. gondii* serum derived from mice 22 days after infection with Pru, and rabbit anti-BAG1 antibody. One hundred anti-*T. gondii*-reactive vacuoles were visually scored using a 40X objective and Zeiss Axiovert 100TV phase contrast microscope with FluoArc lamp and Zeiss filter sets 31, 34, 38 and 50. BAG1 expression was scored as complete if all DAPI-reactive parasites within the vacuole also reacted with anti-BAG1 antiserum.

### Luciferase assay

Luciferase activity was quantified as described [25]. Briefly, intracellular parasites were isolated by passage through a 27 g needle, and placed in black 96 well plates at 2×10^6^/well. D-luciferin was added to 0.25 mg/ml, and luminescence was captured by Xenogen IVIS 200, using a background setting of 6000 photons/sec/cm^2^/sr, medium binning, F/stop = 1, and 5 minute integration time. Data was analyzed using LivingImage v3.2 (Caliper Life Sciences, Hopkinton, MA, USA). Negative control values obtained from strain Pru for each experiment were subtracted from test samples as background.

### Western blot

*T. gondii* proteins from 48 hr tachyzoites or 3 day induced bradyzoites were separated on 10% polyacrylamide gels, transferred to Immobilon-P (Millipore, Bedford, MA, USA) and blocked in 5% skim milk in PBS/0.1% Tween 20. Antibodies used were murine anti-CST1 (provided by L.M. Weiss, Albert Einstein College of Medicine, Bronx, NY), mouse monoclonal antibodies 1DE10 and 3AD6, and rabbit anti-TUB2 to detect β-tubulin as a loading control (provided by L.D. Sibley, Washington University School of Medicine, St. Louis, MO). HRP-conjugated secondary antibodies were used in conjunction with the ECL Plus western blotting detection system and scanning on a STORM860 imager using blue laser excitation, PMT at 900V and 100 mm scale detection (GE Healthcare, Little Chalfont, Buckinghamshire, UK). Densitometric values were acquired using ImageQuant v5.2, using local average background subtraction. Normalized values were calculated as (CST1 or TgRSC8/TUB2)_test sample_ × 100.

### Flow cytometry

Infected monolayers were washed twice with Dulbecco's PBS (DPBS) to remove debris and extracellular parasites. Three-day induced bradyzoites were isolated by serial passage through 27 g and 30 g needles then fixed in DPBS with 2% formaldehyde. Samples were permeabilized for 1 hour in DPBS with 5 mM EDTA, 2% BSA and 0.2% saponin, then stained with 3AD6 monoclonal anti-TgRSC8 and rabbit polyclonal anti-BAG1 followed by donkey anti-mouse IgG AlexaFluor 488 and donkey anti-rabbit IgG AlexaFluor 647 secondary antibodies (Invitrogen, Carlsbad, CA, USA). Samples were briefly fixed in 1% formaldehyde then stored in DPBS with 5 mM EDTA and 0.2% BSA at 4°C until analysis. At least 10,000 events per sample were collected on a BD LSR II flow cytometer (BD, Franklin Lakes, NJ, USA). Compensation and analyses were performed using FlowJo (TreeStar, Ashland, OR, USA).

### Quantitative real time RT-PCR (Q-PCR)

Amplifications occurred in 25 µl volumes using 1 µl cDNA template, 200 nM of each primer (listed in Table S2 as GENEqrtF or R), and iQ SYBR Green Supermix (Bio-Rad, Hercules, CA, USA). Reactions were run using iQ iCycler v3, and conditions included an initial 3 minute 95°C denaturation, followed by 40 repeats of 30 seconds at 95°C and 30 seconds at 60°C. All experiments included duplicate samples and melt curve analysis, and indicated single products (approximately 120 bp) of the expected T_m_ were amplified. Primer efficiencies (E) were determined from triplicate 10-fold serial dilution tests, plotting threshold cycle (Ct) versus log dilution on an increasing linear scale, where E = 10^(1/m)^, and were applied to expression level calculations using the Pfaffl modification approach [26]. Housekeeping genes *TUB1* and *GAPDH* contain paralogs in the *T. gondii* genome. Transcript levels of *TUB1* paralogs TgME49_116400, TgME49_031770 and TgME49_031400, and *GAPDH* paralogs TgME49_089690 and TgME49_069190 were analyzed in tachyzoites and bradyzoites by Q-PCR, and all but the undetectable TgME49_031770 were stably expressed between both stages (data not shown). The published *TUB1* locus (TgME49_116400) and *GAPDH* TgME49_089690 were selected for these studies [20]. The crossing thresholds for all of the loci are shown (Fig. S3B). Test transcript levels were normalized to *TUB1* or *GAPDH* levels using the following formula (E*_housekeeping gene_* ^Ct^)/(E_test gene_^Ct^) × 100. The relative expression of test genes appeared similar when normalized to either locus. Analyses of variation (ANOVA) were performed on resulting data. Significance was defined as *p*≤0.05 for mutant versus vector controls, and mutant versus at least two of three complemented strains for all but the enolase locus. Primers for this locus showed reduced efficiency (E = 1.69) in comparison to those for other loci (E = 1.83-2.01), possibly contributing to inter-experimental variability. As each replicate showed at least a two-fold difference between mutant and vector controls, and between mutant and two to three complements, we declare the data to be significant.

## Results

### C9 is disrupted in the *TgRSC8* locus

**Figure 1:**
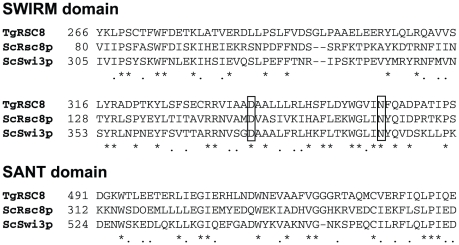
SWIRM and SANT domains of TgRSC8. Alignment of SWIRM and SANT domains of TgRSC8 with yeast Rsc8p (ScRsc8p) and Swi3p (ScSwi3p; Genbank accession numbers NP116695 and NP012359, respectively). At left of the sequence is the first amino acid position number of the regions shown. Asterisks below the alignments indicate sites of amino acid identity, while dots indicate conservative differences. Amino acids in boxes were modified in TgRSC8 in this study.

Screening of a *T. gondii* insertional mutant library identified strains defective in tachyzoite to bradyzoite differentiation in vitro [27]. Identification of the disrupted locus in one of these mutants, strain C9, was performed by plasmid rescue. Sequences flanking the plasmid insertion site were compared to ToxoDB and showed identity to locus *TgME49_086920*. Alignments of the predicted 84 kDa protein using ClustalW showed the TgME49_086920 protein to have 29.4% similarity and 16.5% identity to yeast Rsc8p, and 35.0% similarity and 17.7% identity to yeast Swi3p. Rsc8p and Swi3p both contain SWIRM and SANT domains. The alpha-helical SWIRM domain is predicted to mediate interactions with both protein and DNA in the assembly of chromatin-protein complexes. This domain within Swi3p was shown to directly bind free DNA as well as nucleosomal DNA. Amino acids D374 and N392 were shown to be critical for this process [28]. SWIRM was identified within TgME49_086920 from amino acids 266 to 356 (PFAM-A expect value of 1.6×10^-20^, Pfam v24.0 http://pfam.sanger.ac.uk [29]), sharing key residues with its yeast homologs, including the aforementioned aspartic acid and asparagine (Fig. 1). The SANT domain of Rsc8p was shown to associate with histones, with amino acid W546 critical to the structure [30]. The amino acid R564 in the SANT domain of Swi3p provided a positive charge necessary for function [31]. The SANT domain of TgME49_086920 was located between amino acids 491 and 537 (PFAM-A expect value of 6.8×10^−10^) and showed conservation of the yeast W546 and R564 residues (Fig. 1). As TgME49_086920 was the only Swi3p/Rsc8p homolog identified in the *T. gondii* genome, we named this locus *TgRSC8*.

### C9 produces a polycistronic *TgRSC8* transcript

**Figure 2:**
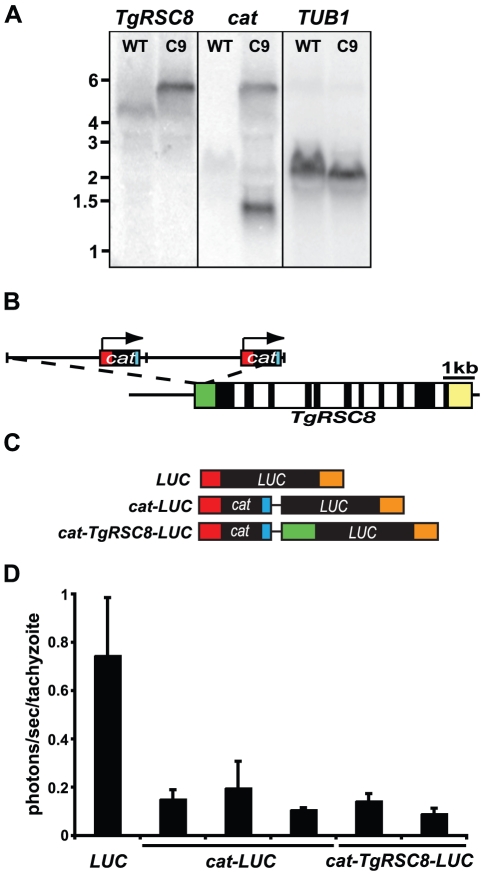
Characterization of the TgRSC8 locus and product. **A.** Northern hybridization indicates *TgRSC8* is affected in C9. Total RNA from PruΔ*HPT* (WT) or mutant C9 was probed with fragments of *TgRSC8* and *cat*. *TUB1* signal was used to assess equivalence of loading. **B.** Map of the insertion site in strain C9. The *TgRSC8* locus is shown with exons (black), introns (white), and 5′ and 3′ UTRs (green and yellow, respectively) to scale. Insertion of 2 copies of pT230-TUB5/CAT (separated by vertical hatch marks) occurred in the 5′ UTR of *TgRSC8*. The *cat* gene and UTRs (red) are indicated on the plasmid cartoon, including the direction of transcription from the *TUB1* promoter (arrow). **C.** Transcripts from luciferase reporter constructs. Maps of possible full-length transcripts from constructs containing a monocistronic *LUC* gene (*LUC*), polycistronic *LUC* encoded downstream of *cat* (*cat-LUC*), and polycistronic *LUC* with a partial *TgRSC8* 5′ UTR (green) between *cat* and *LUC* (*cat-TgRSC8-LUC*) are shown to scale. Additional noncoding sequences, including the *TUB1* 5′ and 3′ UTR (red and orange, respectively), and *SAG1* 3′ UTR (blue) are indicated. Vector sequences are shown as black lines. **D.** Luciferase reporters indicate translation occurs at the downstream locus of a polycistronic transcript in *T. gondii*. Activity is shown for reporter construct transformants whose transcripts are diagrammed in C, and is displayed as photons per second per tachyzoite. Shown is the average and standard deviations of three independent experiments.

Insertion of disrupting plasmid pT/230-TUB/5CAT in C9 occurred upstream of the predicted translational start site of TgRSC8. Southern hybridization results show that the plasmid is present in two copies as a head-to-tail tandem repeat in a single insertion site (data not shown). To determine if insertion occurred within the transcribed region of *TgRSC8*, northern hybridization was performed on total RNA from PruΔ*HPT* and C9 tachyzoites. When probed with a fragment of predicted exon 1 of *TgRSC8* (Fig. S1), a transcript of 4.5 kb was detected in the wild-type strain, while C9 showed a transcript both larger in size and in relative abundance, as determined by probing for the *T. gondii* housekeeping gene encoding α-tubulin (*TUB1*, TgME49_116400) as a loading control (Fig. 2A). Sequencing and rapid amplification of cDNA ends (RACE) performed on the wild-type *TgRSC8* transcript indicated a coding region of 2343 bp, matching the annotation for TgME49_086920, and a 5′ untranslated region of 715 bp. Two products were identified by 3′ RACE, indicating 3′ UTR sizes of 216 bp or 853 bp, the latter of which matches the predicted 3′ UTR on ToxoDB and more closely aligns with the total transcript size detected by hybridization. Plasmid insertion in C9 occurred within the 5′ UTR, 135 bp downstream of the transcriptional start site. Plasmid sequences rescued from C9 indicated that the *T. gondii* selectable marker of pT/230-TUB/5CAT was juxtaposed upstream of the *TgRSC8* open reading frame (ORF). This bacterial-derived 663 bp chloramphenicol acetyltransferase (*cat*) gene is transcribed from a constitutive *T. gondii TUB1* promoter, and employs the *TUB1* 5′ UTR, and *SAG1* 3′ UTR and downstream sequence. When probed with the *E. coli cat* coding sequences, no transcript is detected in PruΔ*HPT*, however, two transcripts are detected in the C9 transformant (Fig. 2A). The *cat* transcript at 1.3 kb corresponds with *cat* expressed from the inserted plasmid, and the second transcript at 5.8 kb matches the size of the transcript detected with the *TgRSC8* probe. These data indicate that in strain C9, transcription of *TgRSC8* originated within the inserted plasmid, creating a fused *cat-TgRSC8* transcript (Fig. 2B). The *cat-TgRSC8* transcript is upregulated relative to the *TgRSC8* transcript alone, likely due to transcription from the constitutive *TUB1* promoter.

### Translation of a polycistronic transcript in *T. gondii*

As *TgRSC8* is a downstream gene on a polycistronic transcript in the C9 mutant, we sought to determine if it was translated in *T. gondii*. A construct was designed in which the *TgRSC8* ORF was replaced by a promoterless firefly luciferase gene (*LUC*), creating a fusion of *cat* and *LUC,* with 580 bp of *TgRSC8* 5′UTR in between (*cat-TgRSC8-LUC,* diagramed in Fig. 2C). Expression of luciferase in two PruΔ*HPT*/*cat-TgRSC8-LUC* transformants was compared to that of a PruΔ*HPT* transformant containing monocistronic *TUB1*-transcribed luciferase. The polycistronic luciferase construct resulted in detectable reporter activity above background (Fig. 2D). However, luciferase activity was approximately 6-fold higher from the monocistronic luciferase construct, suggesting a relative reduction in translation of the downstream ORF. To determine if the partial *TgRSC8* 5′ UTR between *cat* and *LUC* impacted translation efficiency, a direct *cat-LUC* fusion was created. Three independent PruΔ*HPT*/*cat-LUC* transformants showed luciferase activity similar to that of PruΔ*HPT/cat-TgRSC8-LUC* transformants, suggesting no translational contribution from these sequences (Fig. 2D).

### C9 is defective in BAG1 expression

**Figure 3:**
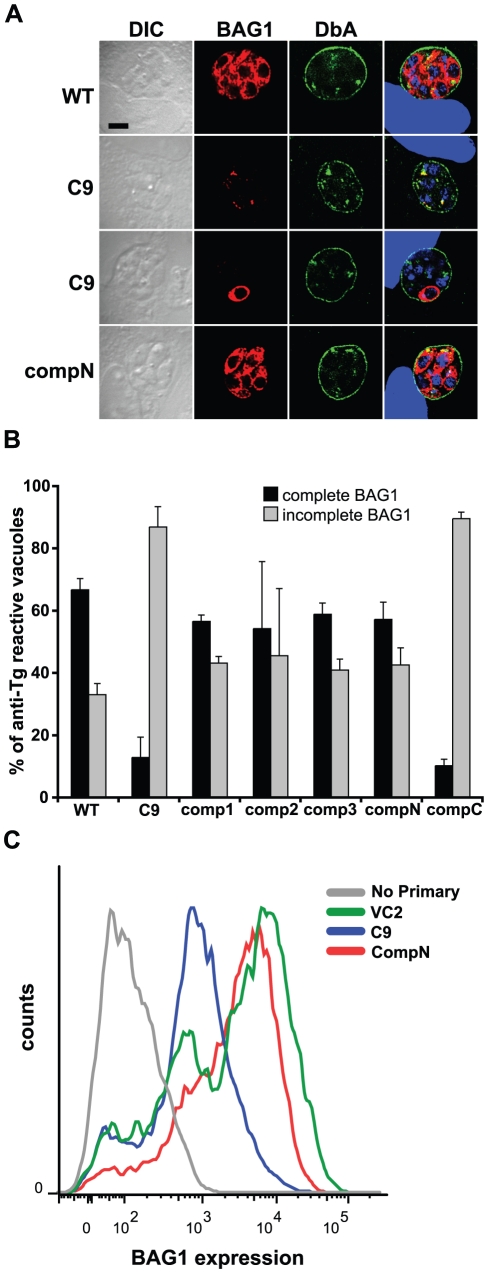
Expression of BAG1 is reduced in strain C9 bradyzoites. **A.** BAG1 expression and reactivity with *Dolichos biflorus* agglutinin (DbA) was analyzed in bradyzoites of strains PruΔ*HPT* (WT), C9 and an amino-terminally tagged, genetically complemented strain (compN) by immunofluorescence microscopy. The left column contains differential interference contrast (DIC) images. The rightmost column contains merged images including DAPI-detection of DNA (blue). All vacuoles show surface reactivity with DbA (green) indicating bradyzoite conversion, but BAG1 expression (red) of C9 is absent, or on only some bradzoites within. **B.** Quantitation of BAG1 expression by fluorescence microscopy. Vacuoles reacting with mouse anti-*T. gondii* (Tg) serum were assessed for BAG1 expression. Those showing anti-BAG1 reactivity of all DAPI-detected bradyzoites within were scored as complete (black bars), while BAG1-negative vacuoles and those containing only some bradyzoites were scored as incomplete (gray bars), displayed as a percentage of anti-Tg reactive vacuoles. Strain C9 shows a reduction in complete BAG1 reactivity relative to WT that is increased on genetic complementation of disrupted locus *TgRSC8* by plasmid pTPR17 (comp1-3). Addition of an amino-terminal HA-tag to TgRSC8 also complemented the BAG1 phenotype (compN), but placement of the tag at the carboxy-terminus does not (compC). Shown is the average and standard deviation of three independent experiments. **C.** Quantitation of BAG1 expression by flow cytometry. Reactivity of C9 to anti-BAG1 antiserum (blue) was compared to that of a vector control strain (VC2, green), and C9 complemented strain compN (red). Background reactivity to fluorescent-conjugated secondary antiserum in the absence of anti-BAG1 antiserum exposure is shown for strain VC2 (gray). BAG1 reactivity is indicated on the x-axis in arbitrary units, and events counted (counts) are on the y-axis. Strains were analyzed in three independent experiments, showing similar outcomes. Results of a representative experiment are shown.

Strain C9 demonstrated a defect in expression of BAG1, but not of cyst wall components reacting with *Dolichos biflorus* agglutinin, both of which are markers traditionally used to identify bradyzoites [27]. To quantify the BAG1 expression phenotype, PruΔ*HPT* and C9 parasites were subjected to bradyzoite induction conditions. Three days post-induction, infected monolayers were fixed and reacted with mouse anti-*T. gondii* antiserum to detect all parasitophorous vacuoles, rabbit anti-BAG1, and DAPI to identify parasite nuclei. BAG1 expression was scored as complete if all DAPI-reactive parasites within the vacuole reacted with anti-BAG1 antiserum. While BAG1 was readily detected in most vacuolar parasites of parental strain PruΔ*HPT*, over 80% of vacuoles of identically treated C9 parasites display incomplete BAG1 expression (Fig. 3A,B). Flow cytometric quantitation of BAG1 in parasites exposed to bradyzoite induction conditions also demonstrated reduced reactivity in the C9 mutant in comparison to vector control-containing parasites (Fig. 3C).

To determine if addition of *TgRSC8* could restore C9 to wild-type levels of BAG1 expression for in vitro developed bradyzoites, we generated a construct containing the *TgRSC8* cDNA, flanked by 2.2 kb of native upstream, and 200 bp of downstream sequence. Three independent C9 transformants were compared to wild-type and C9 parasites for BAG1 expression by immunofluorescence microscopy, three days after bradyzoite differentiation. All three complements restored BAG1 expression to levels characteristic of wild-type *T. gondii*, with the majority of vacuoles demonstrating complete BAG1 reactivity (Fig. 3A,B). Interestingly, fusion of an HA tag to the amino-terminus of TgRSC8 did not impact this complementation, whereas clones containing a HA tag on the carboxy-terminus of TgRSC8 did not complement the BAG1 expression defect of the C9 mutant (Fig. 3B; data not shown). Flow cytometry confirmed the wild-type levels of BAG1 expression in a complemented strain (Fig. 3C).

### CST1 expression is unchanged in C9

As BAG1 expression was affected in the TgRSC8 mutant, we analyzed the expression of another marker of bradyzoite development. Cyst wall glycoprotein CST1 is thought to be in part responsible for reactivity of cysts with *Dolichos biflorus* agglutinin, commonly used to assess *T. gondii* bradyzoite differentiation [32]. A significant change in reactivity with *D. biflorus* agglutinin was not noted in the C9 mutant relative to the wild-type strain (data not shown) [27]. Expression of CST1, whose genomic locus is unknown, was examined in PruΔ*HPT*, C9, and complemented strains by western immunoblot. Not surprisingly, CST1 expression was found to be unaffected in the TgRSC8 mutant (data not shown).

### Generation of antibodies to TgRSC8

**Figure 4:**
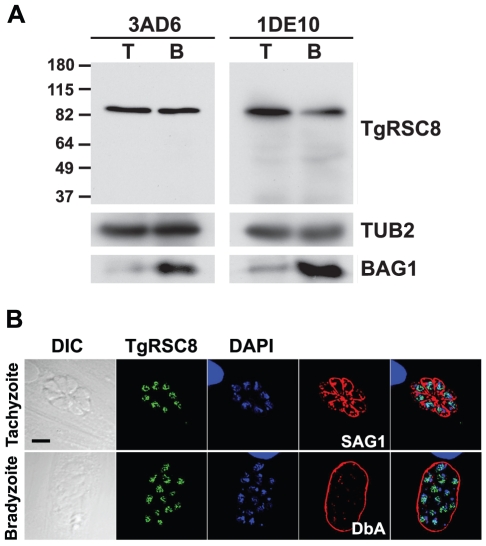
Anti-TgRSC8 antibodies detect a 84kDa nuclear protein. **A.** Anti-TgRSC8 monoclonal antibodies 3AD6 and 1DE10 detect an 84 kDa protein in extracts of either tachyzoites (T) or bradyzoites (B) of the vector control stain VC2 by western immunoblot. Markers of molecular mass are shown at the right in kDa. Blots were reprobed to indicate reactivity of β-tubulin (TUB2) and BAG1 as controls for loading and bradyzoite transition, respectively. **B.** TgRSC8 is a nuclear protein in both tachyzoites and bradyzoites. TgRSC8 was detected in wild-type parasites using 1DE10 antibody (green). DNA is detected by DAPI (blue). Tachyzoites are indicated by detection of the surface marker SAG1, while bradyzoites are shown by *Dolichos biflorus* agglutinin (DbA) reactivity (red). At left are DIC images, including a 5 µm scale indicator (black bar), and merged panels are shown on the right.

To further characterize TgRSC8, monoclonal antibodies were generated against the purified recombinant protein. By denaturing western immunoblot, antibodies 1DE10 and 3AD6 reacted strongly with an 84 kDa protein in both tachyzoite and in vitro developed bradyzoite protein extracts (Fig. 4A). This corresponds to the protein mass based on translation of the predicted ORF. These anti-TgRSC8 monoclonal antibodies were used to examined the subcellular localization of TgRSC8. In *S. cerevisiae,* Rsc8p localizes to the nucleus, and directly complexes to DNA and histones to control gene expression. Using either monoclonal antibody for detection within *T. gondii* tachyzoites and in vitro developed bradyzoites, TgRSC8 appeared in the same compartment with nuclear DNA as identified by both DAPI and reactivity with antiserum against the major *T. gondii* H2B histone, H2Bv (Fig. 4B; data not shown) [33]. Although TgRSC8 was clearly nuclear, it did not co-localize precisely with either DAPI-detected nuclear DNA, or H2Bv. To confirm the specificity of the monoclonal antibodies and localization of TgRSC8, the complemented C9 transformant expressing amino-terminal HA tagged TgRSC8 was assessed using anti-HA antibody-based detection (Fig. 3). As this HA tagged version of TgRSC8 complemented the C9 mutant, this suggests correct localization of the protein. Amino-terminal HA tagged TgRSC8 also localized to the parasite nucleus in a pattern similar to that seen using the monoclonal antibodies against TgRSC8 (Fig. S2). While addition of the HA tag to the carboxy-terminus of TgRSC8 rendered it non-functional with regard to the effects on BAG1 expression (Fig. 3B), it also localized to the nucleus in both tachyzoites and in vitro bradyzoites (data not shown). These results demonstrate the specificity of the monoclonal antibodies for detection of TgRSC8, which appear within the nucleus of *T. gondii*.

### TgRSC8 protein is reduced in C9

**Figure 5:**
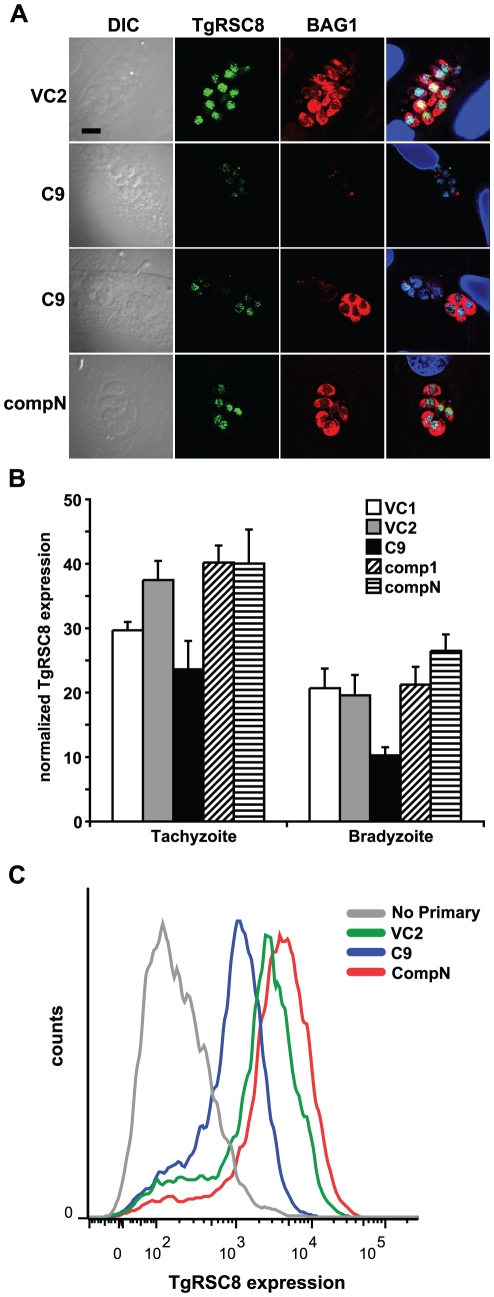
TgRSC8 expression is reduced in C9. **A.** Expression of TgRSC8 by vector control stain VC2, strain C9, and a C9/TgRSC8 complement (compN) was analyzed using fluorescence microscopy. Reactivity with monoclonal anti-TgRSC8 antibody 1DE10 is shown in green and rabbit anti-BAG1 reactivity in red. Images include representatives of the varying phenotype of C9, including a vacuole in which neither TgRSC8 nor BAG1 is detected (upper). Another C9 image (lower) shows adjacent vacuoles, one in which parasites are expressing BAG1 but not TgRSC8, and another showing one anti-TgRSC8-reactive parasite, but none expressing BAG1. At left are DIC images, and a bar indicating 5 µm scale is shown. At right are merged images including DAPI detection of DNA, shown in blue. **B.** TgRSC8 expression from vector control strains VC1 (white bars) and VC2 (gray bars), C9 (black bars), and C9/TgRSC8 complements (comp1 and compN, hatched bars) was quantified by western immunoblot in both tachyzoite- and bradyzoite-stage parasites. TgRSC8 levels were normalized to the levels of β-tubulin per sample, and TgRSC8 expression is expressed as a percentage of β-tubulin expression. Shown are the averages and standard errors of three independent experiments. Student's *t*-tests indicate differences between C9 and vector controls, and C9 and complements are significant (*p*≤0.05) for all but C9 versus VC1 in tachyzoites. **C.** Quantitation of bradyzoite TgRSC8 expression by flow cytometry. TgRSC8 was detected in cultures exposed to bradyzoite induction conditions by anti-TgRSC8 monoclonal antibody reactivity. Strains analyzed include mutant C9 (blue), a vector control strain (VC2, green), and C9 complemented strain compN (red). Reactivity to fluorescent secondary antiserum in the absence of primary antibody is shown for strain VC2 (gray). TgRSC8 reactivity is shown on the x-axis in arbitrary units, and the y-axis indicates counted events (counts). Strains were analyzed in three independent experiments, which demonstrated similar results. A representative experiment is shown.

**Figure 6:**
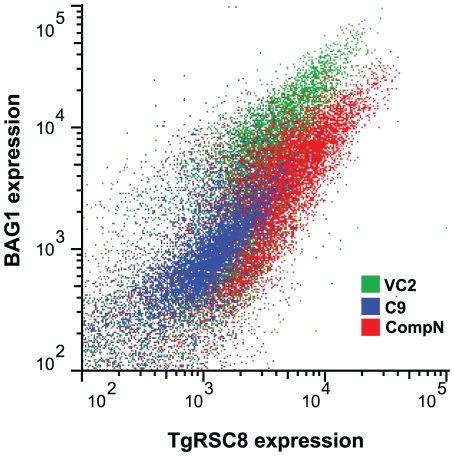
TgRSC8 expression correlates with BAG1 expression. TgRSC8 and BAG1 levels were determined for C9 (blue), a vector control strain (VC2, green) and C9 complement compN (red) parasites subjected to bradyzoite induction conditions, by flow cytometry. TgRSC8 levels are displayed on the x-axis and BAG1 levels on the y-axis, in arbitrary units. Three independent experiments were performed, showing similar results. Results of a representative experiment are shown.

In the C9 mutant, *TgRSC8* is the downstream gene on a polycistronic transcript, and thus it may show reduced translational efficiency. Additionally, complementation of the C9 mutant with TgRSC8 restored BAG1 expression to wild-type levels. These data suggested a reduction in expression of TgRSC8 in C9 as compared to the wild type. To facilitate quantification of TgRSC8, we used the 3AD6 monoclonal antibody. A reduction in TgRSC8 in C9 was detected by immunofluorescence using monoclonal anti-TgRSC8 antibody (Fig. 5A). To quantify the reduction in TgRSC8 protein in the C9 mutant, vector control strains containing disrupting plasmid pT/230-TUB/5CAT were compared to C9 and complemented mutants by western immunoblot and flow cytometry, examining populations of both tachyzoites and in vitro bradyzoites. TgRSC8 was reduced in populations of both stages of C9 parasites relative to either vector controls or complements (Fig. 5B,C; data not shown). Western blots, flow cytometry, and immunofluorescence microscopy revealed that this reduction was minimal though detectable in C9 tachyzoites, whereas the reduction of TgRSC8 in C9 bradyzoites was more pronounced (Fig. 5B; data not shown). Complementation with the *TgRSC8* locus increased expression of this protein in both tachyzoites and bradyzoites over that of the mutant C9 (Fig. 5A,B,C; data not shown). The level of TgRSC8 in bradyzoites correlated with expression of BAG1 in strain C9. Parasites showing a loss of BAG1 expression similarly demonstrated a loss of TgRSC8, though some BAG1 positive C9 parasites were also found to have reduced TgRSC8 expression, a variability not seen in BAG1 positive parasites of the vector control strain (Fig. 5A). Flow cytometric analyses of C9, a C9 complement and vector control bradyzoites using 3AD6 ascites showed that increased TgRSC8 expression was associated with increased BAG1 expression for all strains tested (Fig. 6). Taken together, these data demonstrate that despite increased levels of transcript, insertion within the 5′UTR of *TgRSC8* resulted in a reduction in translated protein, and that TgRSC8 facilitates wild-type expression of BAG1 from in vitro bradyzoites.

### Substitution variants of TgRSC8

Yeast Swi3p with alanine substitutions of amino acids D374 and N392 was expressed and assembled into SWI/SNF complexes, but it did not bind DNA [28]. To assess TgRSC8 functionality, the amino-terminal HA-tagged complementation construct was modified such that the corresponding amino acids, D337 and N355, were substituted with alanine singly and in combination. No viable cells containing D337A TgRSC8 transforming DNA were recovered from multiple electroporations of *T. gondii* strain C9. A single transformant each of N355A and double substitution clones were obtained from multiple electroporations, as determined by Southern hybridization. Although transcript was evident by RT-PCR, these clones did not express detectable substituted TgRSC8 by anti-HA western blot (data not shown). As TgRSC8 is expressed in C9, it is possible that substituted TgRSC8 acts in a dominant negative fashion, leading to inviability of recipient parasites and selection for non-expressing clones.

### Reduced expression of TgRSC8 affects gene expression in *T. gondii*

**Figure 7:**
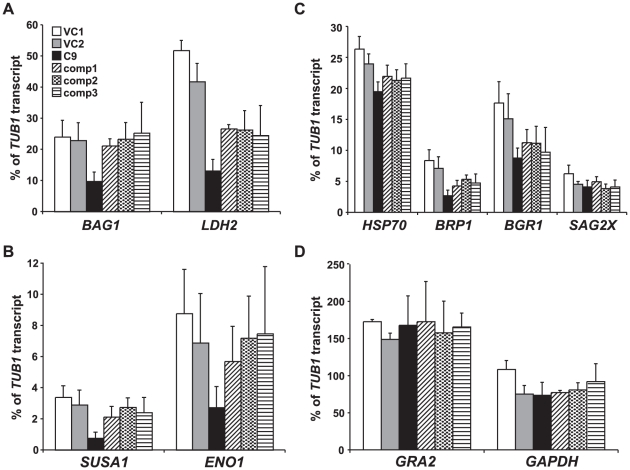
Effects of TgRSC8 reduction on transcript levels of bradyzoite-induced and constitutive genes. Q-PCR was performed on cDNA derived from in vitro bradyzoites of vector control strains VC1 (white bars) and VC2 (gray bars), TgRSC8 mutant C9 (black bars), and three complemented strains (comp1-3, patterned bars) to analyze transcript levels of bradyzoite-induced genes *BAG1*, *LDH2* (panel **A**), *SUSA1*, *ENO1* (panel **B**), a bradyzoite-induced *HSP70*, *BRP1*, *BGR1* and *SAG2X* (panel **C**). Transcripts of constitutive genes *GRA2* and *GAPDH* were also quantified (panel **D**). Transcript levels were expressed as a percentage of the level of housekeeping gene *TUB1* per sample. Shown are the averages and standard deviations of three experiments, performed on cDNAs derived from independent RNA samples.

*S. cerevisiae* Rsc8p is known to impact transcription of many genes in yeast. As BAG1 protein expression in C9 was reduced in bradyzoites, the steady state levels of several transcripts previously described as induced in bradyzoites, as well as two constitutively transcribed genes, were determined for strain C9, three complemented strains and two vector control strains by Q-PCR in bradyzoites. The genes analyzed included bradyzoite-induced genes encoding BAG1 (TgME49_059020), LDH2 (TgME49_091040), SUSA1 (TgME49_078080), ENO1 (TgME49_068860), SAG2X (SRS49B, TgME49_007140), BRP1 (TgME49_114250), and BGR1 (TgME49_053330), which represent a diverse group from metabolic enzymes to surface markers [34-38; Buchholz and Boothroyd, personal communication]. As BAG1 is a heat shock protein, we sought an additional heat shock protein target for study. Analysis indicating *T. gondii* expresses a bradyzoite-induced HSP70 was performed using methods now known to detect three closely related HSP70s (TgME49_073760, _111720, and _051780) [39]. Locus TgME49_111720 showed the largest number of bradyzoite expressed sequence tags, and was selected for analysis (ToxoDB). Transcript levels of housekeeping genes *TUB1* and *GAPDH*, and the gene encoding dense granule protein 2 (GRA2, TgME49_027620), predicted to be expressed equivalently in both stages in wild-type *T. gondii*, were also assessed (Fig. S3A; ToxoDB) [40]. In keeping with microscopic evidence of BAG1 protein expression, the *BAG1* transcript was decreased in the *TgRSC8* mutant and restored to the levels of the control strains in complements (Fig. 7A). Steady-state transcript levels of the bradyzoite-induced genes *LDH2*, *SUSA1* and *ENO1* transcripts were also significantly reduced in the mutant relative to vector controls, and increased in complemented strains (Fig. 7A,B). No effect was seen on transcript levels of *GAPDH*, or *GRA2*, which were equivalently expressed in both tachyzoites and bradyzoites (Fig. 7D; data not shown).

Other bradyzoite-induced genes showed a different profile of expression in the strains tested. The transcript levels of surface marker SAG2X and bradyzoite-specific dense granule protein BGR1 were not affected in the *TgRSC8* mutant, while those of the assessed heat shock protein and bradyzoite-specific rhoptry protein BRP1 were reduced in C9, but not significantly increased in the complemented strains (Fig. 7C). Interestingly, loci from which transcript levels were restored upon introduction of additional TgRSC8 to C9 showed higher levels of induction after in vitro bradyzoite differentiation as compared to the other loci. *BAG1*, *ENO1*, *LDH2* and *SUSA1* were 736-, 230-, 144- and 142-fold upregulated in bradyzoites as compared to tachyzoites. In contrast, *BRP1*, *BGR1*, *SAG2X* and the examined *HSP70* locus showed more modest inductions of 33-, 28-, 28-, and 1.4-fold, respectively (data not shown).

## Discussion

Despite its position as the second gene in a polycistronic mRNA, *TgRSC8* is translated in the C9 mutant. Similarly, luciferase activity was detected from a promoterless *LUC* gene placed downstream of *cat* when within the same sequence context as *TgRSC8* in strain C9. While translation of downstream genes does occur in polycistronic transcripts, it is surprising that the non-native message generated by plasmid insertion would create a dicistronic message that's downstream gene is translated. Many transcripts in *T. gondii* have lengthy 5′ untranslated regions, suggesting this parasite is capable of initiating translation far from the cap site of the message. Removal of intervening sequences between the *LUC* and *cat* genes did not impact LUC expression, indicating the lack of an internal ribosomal entry site, or other specific sequence elements to recruit translational machinery downstream of *cat*. TgRSC8 translation in the C9 mutant must occur either by the translational machinery bypassing a start site within reasonable context to initiate at a downstream site, or the translational machinery must not disengage at the stop codon of *cat* to allow translation of the downstream ORF. A consensus translation initiation sequence of gNCAAaATGg was identified in *T. gondii*, shown with the start codon underlined [41]. In the polycistronic *TgRSC8* message, the *cat* ORF is translated from the first methionine, and has the more favorable context of GACAAAATGC. However, the context of the start codon of *TgRSC8* in C9 is not as favorable, but is identical to the native locus, and is GCCGCGATGT. The translation of *TgRSC8* in C9 will be a topic of future study.

We show that a suspected component of the ATP-dependent chromatin remodeling system, TgRSC8, facilities the expression of some bradyzoite-induced genes, including *BAG1*. Cis-acting sequences were identified within the *BAG1* promoter that were sufficient to induce bradyzoite-specific expression when placed within a constitutive promoter [5]. Additionally, histone analysis at the *BAG1* promoter determined that acetylation states correlate with stage-specific expression. These data suggest the cooperative function of traditional cis-acting sequence elements with both histone-modification and nucleosome remodeling arms of epigenetic control. The intersection of histone-modification and chromatin-remodeling machinery has been uncovered previously in *T. gondii* as the histone methylation activities of TgCARM increased in the presence of ATP, suggesting a linkage with ATP-dependent chromatin remodeling [9]. The mechanism of recruitment of RSC complexes to specific promoters is not fully understood, but three theories were proposed [42]. One model suggests recruitment by direct association with the RNA polymerase II holoenzyme, as has been shown for SWI/SNF, while another relies on recruitment by gene-specific transcriptional activators. A third model suggests the induction of transient chromatin changes throughout the genome, which are stabilized to alter expression in the presence of DNA-binding transcription factors. This third model may be the most accurate as efforts to prove direct association of TgRSC8 with DNA or transcription-related proteins was unsuccessful and thus the effect may be indirect (data not shown). The *BAG1* promoter may require the presence of a trans-activating factor to recruit ATP-dependent remodeling machinery or to stabilize chromatin changes, alleviating nucleosomal repression.

The RSC and SWI/SNF yeast chromatin remodeling complexes share conserved components, including Rsc8p and Swi3p. These conserved components were proposed to comprise the structural core of their respective complexes [43]. While other complex components are present in the *T. gondii* genome, only TgRSC8 contains both SWIRM and SANT domains, and no paralogs were found within the genome. *T. gondii* may not contain multiple conserved remodeling complexes as are found in yeast, or alternatively, TgRSC8 could associate with multiple distinct complexes. Attempts at generation of a *TgRSC8* knockout in wild-type *T. gondii* were unsuccessful, indicating this may be an essential gene (data not shown). The inability to derive D337A and N355A TgRSC8 variants suggests that these residues play an important role in functionality or stability of TgRSC8. Expression of dysfunctional copies of TgRSC8 in tachyzoites may impair the function of existing copies of TgRSC8 in strain C9, as yeast Rsc8p forms dimers [44]. Both of these results highlight a role for TgRSC8 in tachyzoite stage parasites.

Housekeeping genes *TUB1*, *GAPDH*, and constitutively expressed *GRA2* were unaffected by the TgRSC8 reduction found within strain C9. This suggests either a lack of necessity for TgRSC8 function at these loci, or that fewer TgRSC8 molecules are required to mediate their transcription than are required for bradyzoite-induced *BAG1*, *LDH2*, *ENO1* and *SUSA1*. Genes encoding a bradyzoite-upregulated HSP70 and BRP1 showed a reduction in bradyzoite transcription in the mutant, yet were not significantly increased by introduction of additional TgRSC8. This and the lack of impact of TgRSC8 reduction on other bradyzoite-induced loci may also be due to the dosage of this molecule found in strain C9. Additionally, the level of TgRSC8 protein was reduced in bradyzoites relative to tachyzoites in wild-type strains. This suggests TgRSC8 may act as a negative regulator of transcription of bradyzoite-induced genes in tachyzoites. In this case, decrease of TgRSC8 during bradyzoite development may trigger transcription of bradyzoite-induced genes. However, complementation of the C9 mutant by addition of *TgRSC8* restored expression of BAG1, inconsistent with this hypothesis. The purpose of reduction of TgRSC8 during in vitro bradyzoite development remains unclear, but may be involved in the relative timing of the cascade of gene expression during bradyzoite development. These results provide further evidence that bradyzoite gene expression in *T. gondii* is a complex process governed by multiple mechanisms.

## Supporting Information

Figure S1 ***TgRSC8*** **primers and probe.** The *TgRSC8* locus is shown with boxed exons (black), introns (white), and 5′ and 3′ UTRs (gray) to scale. Plasmid insertion in strain C9 occurred in a NotI site within the 5′ UTR of *TgRSC8* (lollipop). Positions of primers used for generation of complementation constructs are indicated by small arrows (see also Table S1). A probe used in northern hybridization, representing the first exon of *TgRSC8*, is indicated as a black bar.
(EPS)

Figure S2 **HA-tagged TgRSC8 is a nuclear protein in both tachyzoites and bradyzoites.** Amino-terminally HA-tagged TgRSC8 was detected in strain compN by anti-HA reactivity (green). DNA is detected by DAPI (red). Tachyzoites are indicated by detection of the surface marker SAG1, while bradyzoites are shown by DbA reactivity (blue). At left are DIC images, including a 5 µm scale indicator (black bar), and merged panels are shown on the right.
(EPS)

Figure S3 **Analysis of steady-state transcript levels by Q-PCR.** **A.** Transcripts corresponding to housekeeping genes *TUB1* and *GAPDH* were assessed in both tachyzoites (gray bars) and in vitro-developed bradyzoites (black bars) of wild-type strain PruΔ*HPT*. The threshold cycles for transcript detection are indicated on the y-axis. Shown are the averages and standard deviations of three experiments performed on independent samples. **B.** Effects of TgRSC8 reduction on Q-PCR threshold cycles representing transcript levels of bradyzoite-induced and constitutive genes. Q-PCR was performed on cDNA derived from in vitro bradyzoites of vector control strains VC1 (dark blue bars) and VC2 (yellow bars), TgRSC8 mutant C9 (red bars), and three complemented strains (comp1-3; green, light blue and orange bars, respectively). Threshold cycle values for transcript detection are on the y-axis. Shown are the averages and standard deviations of three experiments, performed on cDNAs derived from independent RNA samples.
(EPS)

Table S1 (DOC)

Table S2 (DOC)
