# Supplementary material for: Involvement of a Toxoplasma gondii Chromatin Remodeling Complex Ortholog in Developmental Regulation
Source: PLoS One. 2011 May 31;6(5):e19570. doi: 10.1371/journal.pone.0019570 (PMC3104990; doi:10.1371/journal.pone.0019570)
Supplement: Table S1 — (DOC) [file pone.0019570.s004.doc]

**Table S1. Primer sequences for cloning and probe generation**

| **Name** | **Sequence (5’ to 3’)** |
| --- | --- |
| RSC8probeF | ATGTCGAATGAGGACGCAATACCTC |
| RSC8probeR | TGGCGACGTTCGGGGCGATTTCTACAG |
| CATprobeF | GATGTGGCGTGTTACGGTGAAAACCTG |
| CATprobeR | ATCCCCCTCGGGGGGGCAAGAATTGTG |
| P1 | CCTGCGAGAGGACAGTGCCTGTTCCCAGACACTG |
| P2 | CGCCTCGGCTGTCTCGACAC |
| P3 | GGAGCCTCTGGCGAAGAC |
| P4 | TTAATTAAGGCAGCGCCGTCAGCGCAG |
| P5 | AATTAATTAACCTCGGCTCTGCTTGCGACAGAA |
| P6 | TTAATTAAGTTTAAACTGTAGATCTGTAAGTCTC |
| P7 | GTGTCTGGGGAGAAGGGAGAGTATCCGA |
| P8 | CCTTAATTAAATCGCGTAGTCTGGGACGTCGTATGGGTAAGGCAGCGCCGTCACGCAGCTTC |
| P9 | GGTCTCATTCTCGCTCGACAACGCAG |
| P10 | GACGTCGTATGGGTACGCCATCGCGGCATGCGACGAGAG |
| P11 | ATGGCGTACCCATACGACGTCCCAGACTACGCGTCGAATGAGGACGCAATACCTC |
| P16 | TCTAGACTTTGATATGCATGTCCGCGTTCGTG |
| P17 | GTTTTTGGCGTCTTCCATCGCGGCATGCGACGAGAG |
| P18 | CTCTCGTCGCATGCCGCGATGGAAGACGCCAAAAAC |
| P19 | TCTAGACGAAGTCATTGTGTGGCACACATC |
| P20 | GTTTTTGGCGTCTTCCATAGTGGATCCCCCTCGGGG |
| P21 | CCCCGAGGGGGATCCACTATGGAAGACGCCAAAAAC |
| P22 | ACCATGGCGAATGAGGACGCAATACCTC |
| P23 | GCGGCCGCAGGCAGCGCCGTCAGCGCAGCTTC |
