# Supplementary material for: Involvement of a Toxoplasma gondii Chromatin Remodeling Complex Ortholog in Developmental Regulation
Source: PLoS One. 2011 May 31;6(5):e19570. doi: 10.1371/journal.pone.0019570 (PMC3104990; doi:10.1371/journal.pone.0019570)
Supplement: Table S2 — (DOC) [file pone.0019570.s005.doc]

Table S2. Primer sequences for Q-PCR

| **Name** | **Sequence (5’ to 3’)** |
| --- | --- |
| TUB1qrtF | GACGACGCCTTCAACACCTTCTTT |
| TUB1qrtR | AGTTGTTCGCAGCATCCTCTTTC |
| GAPDHqrtF | TGGTGTTCCGTGCTGCGATGGAAC |
| GAPDHqrtR | GAGCTTGCCGTCCTTGTGGCTGAC |
| GRA2qrtF | GACGTGCCTTTCAGCGGTAAAC |
| GRA2qrtR | CCGGTTCTTCTGGCTCTTGTTG |
| BAG1qrtF | GATGACGTAACCATAGAAGTCGACAAC |
| BAG1qrtR | GCAAAATAACCGGACACTCGCTCAGTC |
| LDH2qrtF | GGAACCATGGGCTACCTTTGTG |
| LDH2qrtR | CTCATACTGGTTTGCGCTCGTCAC |
| ENO1qrtF | GACATCGTTGCACGCGAGATTTTG |
| ENO1qrtR | AGAGCCTCATAAATACCCGTAGAC |
| SUSA1qrtF | GCATTAGTGGAAGCAAATACCATAAG |
| SUSA1qrtR | TGTCCTGCTCCACATGCAAA |
| SAG2XqrtF | AGCCAACTGAAGATGGGAAACTC |
| SAG2XqrtR | ACTCTGTATCTGGGGTAGAGTTGTTC |
| BGR1qrtF | GCAATTTCCTTCTTTACGGAGAAC |
| BGR1qrtR | GTAAGACATCCACGACCAGAAAG |
| BRP1qrtF | ACGCGAAAGACGGTGTGAAAG |
| BRP1qrtR | TCTGCCTCCTTCTGTAGCTTGTCT |
| HSP70qrtF | CTGTCGCTGCCAGTGATGCGGAGGAAG |
| HSP70qrtR | ATGATGTCAACGCGGCCATGGCGGTAC |
